# Exotic Pet Trade as a Cause of Biological Invasions: The Case of Tree Squirrels of the Genus *Callosciurus*

**DOI:** 10.3390/biology10101046

**Published:** 2021-10-15

**Authors:** Maria Vittoria Mazzamuto, Lucas A. Wauters, John L. Koprowski

**Affiliations:** 1 Haub School of Environment and Natural Resources, University of Wyoming, 1000 E. University Ave, Laramie, WY 82071, USA; jkoprows@uwyo.edu; 2 Department of Theoretical and Applied Sciences, University of Insubria, Via J.H. Dunant 3, 21100 Varese, Italy; lucas.wauters@uninsubria.it

**Keywords:** invasive species, Pallas’s squirrel, Finlayson’s squirrel, bark-stripping, eradication cost, sociopolitical support, economic damage, parasite release, interspecific competition, taxonomy

## Abstract

**Simple Summary:**

The pet industry is a growing global multibillion dollar market. The increase of exotic and non-domesticated animal ownership has led to an increase in the number of non-native pets released that create invasive alien species (IAS) populations in the wild. IAS negatively impact the biodiversity, human health and countries’ economies. We use tree squirrels of the genus *Callosciurus* as a well-documented case study of pets that become IAS. We review the pathways and range of introduction and the challenge and legal importance of species identification. Next, we document how they negatively affect native plants and animals, their parasitic infections that can threat native wildlife and human health and their impact on human activities and productive systems. We discuss the diverse biological, social, political and economic reasons that make control/eradication of these charismatic species difficult in most countries. However, we also highlight the successful management of the IAS in two countries where the early detection and engagement of stakeholders were key to successful eradication. We conclude that efforts to educate and involve the broader public by actively engaging a diversity of stakeholders are more likely to build a consensus toward IAS management and should be a priority for each country.

**Abstract:**

The trade of non-native pets, especially of non-domesticated and exotic animals, and their subsequent release and establishment of populations is one of the major pathways of introduction for invasive alien reptiles, amphibia, birds and mammals. Here, we use a group of arboreal mammals, tree squirrels of the genus *Callosciurus*, as a well-documented case study, reviewing the pathways of introduction, the current areas of non-native distribution, the rate of establishment success and the challenge and legal importance of species identification. We further illustrate the importance of early detection and effective monitoring methods and plans. Next, we document how they interfere with native species, their risk of acting as vectors for emerging infectious diseases and their potential role in maintaining parasitic infections that can affect human health. We conclude by reviewing the current management, or the lack of it, and highlight the diverse biological, social, political and economic reasons that make control/eradication of these charismatic species difficult or even impractical in most countries. However, reviewing the only two successful eradications of the IAS, we highlight the need to acknowledge the public opinion and the importance of communication, transparency and the engagement of a diversity of stakeholders to create a consensus about the actions to undertake.

## 1. Introduction

The pet industry is a global multibillion dollar market growing year after year [1,2,3], now even more with the recent improvement of the living standards in some regions of the world [4,5]. The American Pet Product Association and the European Pet Food Industry estimate that 70% of U.S. and 38% of European households own at least one pet. While cats and dogs account for the majority of pets, 24% of households in USA host fish, ornamental birds, small mammals and reptiles [1]; the trend is similar in Europe, with 35% of pets belonging to the previously mentioned taxa [2]. In fact, over the last decades, the non-native pet trade, live animals transported beyond their native range for sale, has dramatically increased [6,7] thanks to the high revenue of the business, in particular, for some taxa (e.g., birds [8] and reptiles [9,10]). While some traded species have been domesticated for hundreds or thousands of years, wild exotic pets, mostly coming from regions of the world with high species richness, are becoming more and more prominent in the pet trade market [7].

However, the increase of non-native animal ownership has led to an increase in the number of non-native pets released and the subsequent establishment of populations of invasive alien species (IAS) [6,11]. Gippet and Bertelsmeier [12] found that vertebrate IAS are strongly overrepresented in trade, on average being 7.4 times more frequent in trade than in the global species pool. This seems to be related to the fact that trade favors species whose ecological characteristics are associated with invasiveness (e.g., generalist species and large range sizes [6,12]). Several studies have shown how the escape and release of non-native pets is the primary source of vertebrate invasions in many countries (e.g., mammals in Brazil [13] and amphibians and reptiles in the USA [14] and EU [15,16]). Based on the Global Invasive Species Database and the Inventory of Alien Invasive Species in Europe, 53% of invasive vertebrate species have been introduced by the pet trade [17]. The pathway from pet trade to free-ranging animal is by escape, when the owner (importer, seller or consumer) fails to keep the individual secured in captivity, or through release, when the owner intentionally allows the individual to become free-living [17]. Several factors are involved in the reasons for the release of non-native pets; they are mostly related to the biology of the species (e.g., longevity and body size; long-living, large pets are more likely to be released) and socioeconomic factors (number of individuals in the pet market, cost of the pet and cost of the care; commonly sold less expensive species are more likely to be released, as well as those that require expensive long-term care) [6,18,19,20]. There may also be the risk of non-native pets being released into the wild or in urban areas after the entry of new regulations that ban the holding of the newly illegal species [21].

Non-native pets that establish viable IAS populations once released have been associated with negative impacts on the biodiversity, human health and countries’ economies [22,23,24,25]. For example, the common slider, *Trachemys scripta*, is one of the most popular and common pet reptiles introduced in the world [26]. Outside its native range, it becomes invasive and heavily affects native turtle populations (competition) and other invertebrate and vertebrate species (predation) [22,26]. In Brazil, the common marmoset *Callithrix jacchus* threatens the vulnerable native buffy-tufted marmosets *C. aurita* through hybridization [27]. However, as previously mentioned, non-native invasive pets can also directly affect human health, such as, for example, the raccoon *Procyon lotor,* introduced in Europe, which is a rabies vector [28,29], or impact economic activities, for example, the stripping of tree bark by the Eastern grey squirrel *Sciurus carolinensis* on the British Isles, which reduces the value of tree crops and alters forest compositions [30,31].

Tree squirrels are a good example of IAS introduced worldwide mainly through the international pet trade [32,33,34]. Eighteen squirrel species have been introduced in 23 countries over five continents so far [32,35]. Their biological and ecological characteristics have made them successful invaders: broad native distributions worldwide, high vagility and diverse food habits and an elevated capability to establish viable populations from only a few founders [32,36]. Moreover, initial introductions are often followed by illegal translocations in the non-native range, enhancing their ability to spread in new areas [37,38]. The most (in)famous invasive squirrel around the world is the Eastern grey squirrel, known for its spreading ability and its negative impact on native species and the environment [30,39]. Competitive exclusion through competition for food [40,41,42] and parasite-mediated competition are the main mechanisms that affect the survival and persistence of native red squirrel (*S. vulgaris*) populations in Europe [39,43].

While the case of the Eastern grey squirrel has received wide scientific and media attention [44], in the last decade, new studies have been carried out on Asiatic tree squirrels of the genus *Callosciurus* introduced outside their native range. An initial review of their introduction worldwide by Bertolino and Lurz [45] highlighted the possible threats that these squirrels might pose to native plants, animals and humans. Reviewing the recent literature, we will present the data and information gathered on this genus as a case study on how international pet trade and biological invasions can affect ecosystems, native species and human activities.

## 2. The Genus *Callosciurus*

*Callosciurus* Gray, 1867, is a genus of diurnal arboreal squirrels native to Southeast Asia [46], commonly known as the ‘beautiful squirrels’. To this genus belong 16 species very diverse in their morphology, whose identification was originally based on their pelage color and patterns and skull shape [47,48,49]. Among them, Pallas’s squirrel (*Callosciurus erythraeus* Pallas, 1779) and Finlayson’s squirrel (*C. finlaysonii* Horsfield, 1823) are the ones with greater morphological variability [46,50] and are also the ones that have been introduced outside their native ranges [45,51].

*Callosciurus erythraeus* and *C. finlaysonii* are divided into 27 and 16 subspecies, respectively [48,50,52]; however, since their classification was mostly based on morphological characteristics, 18 subspecies of *C. erythraeus* were previously assigned to different species (e.g., *C. flavimanus* is now assigned to a subspecies of *C. erythraeus* [47,48,53]). More recent studies, thanks also to the use of integrative taxonomy, have highlighted the need for a taxonomical revision of the genus [54,55,56].

## 3. Distribution of IAS and Pathways of Introduction

### 3.1. Native Range

Pallas’s squirrel is native to Southeast China (Taiwan included), Laos, Vietnam, Cambodia, Malaysia (mainland), Thailand, Myanmar, Bangladesh, Northeastern India and Bhutan [46,50]. Due to its wide distribution, it can be found from sea-level habitats to subtropical forests to subalpine conifer forests [57]. Finlayson’s squirrel is native to Laos, Vietnam, Cambodia, Thailand and Myanmar [46,50]. In its native range, it typically occurs in subtropical forests and plantations [58]. Both species are tolerant to habitat degradation and fragmentation [59,60].

### 3.2. Areas of Introduction

**Figure 1:**
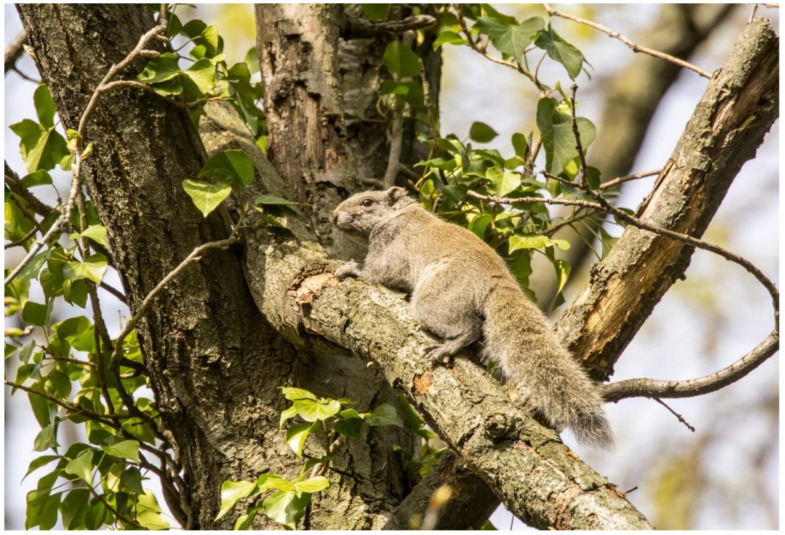
Pallas’s squirrel, *Callosciurus erythraeus*, in Laveno Mombello, Varese Province, Italy. Photo donated by a private citizen to Prof. Dr. Adriano Martinoli of the University of Insubria (Varese, Italy).

Bertolino and Lurz [45] reviewed 28 introduction events and within-country translocations of Pallas’s squirrel in Argentina, Belgium, France, Hong Kong, Japan and the Netherlands. These introductions/translocations led to 20 established populations, 4 unsuccessful establishments and 2 initially established populations but rare since 2011. In one introduction event in Japan, the released individuals were soon removed, and in Belgium and the Netherlands, the populations were eradicated in 2011 and 2015, respectively [61,62]. New studies showed the presence of a new population in Japan in Iruma City (Saitama Prefecture), first detected in 2011 [63], and a more recent one on Mt. Kirishima on the border between Miyazaki and Kagoshima Prefectures, Kyushu, Southwestern Japan [64]. In Argentina, the population previously reported in the city of Buenos Aires [65] failed to persist [66], but several new translocation events have been recorded in the province of Buenos Aires and Santa Fè, raising the number from five releases (one introduction plus four translocations) recorded in 2010 [65] to 28 (one introduction plus 27 translocations) [67]. Moreover, four more translocations of squirrels captured from two of those 27 sites occurred, but the squirrels have been kept in captivity [67]. In France a second population was probably created from individuals purchased on the Internet, then released or escaped from captivity at the beginning of the 2000s in the Department of Bouches-du-Rhône in the town of Istres [68]. The introduction initially attributed to *Callosciurus* sp. in Northern Italy [45] has now been confirmed as *C. erythraeus* [55] (Figure 1). As in Japan and Argentina, also in Italy some illegal translocations have been recorded. In 2021, an individual was observed in an urban park in the city of Varese and another in the village of Magadino in Switzerland (this single squirrel was captured and euthanized), respectively, 21 and 20 km in a straight line from the population established in Northern Italy (Martinoli & Wauters, pers. comm.). Table 1 summarizes the current situation of the invasive Pallas’s squirrel in the world with a total of 56 release events (new introductions and translocations), of which 73% led to established populations (but two populations eradicated).

**Figure 2:**
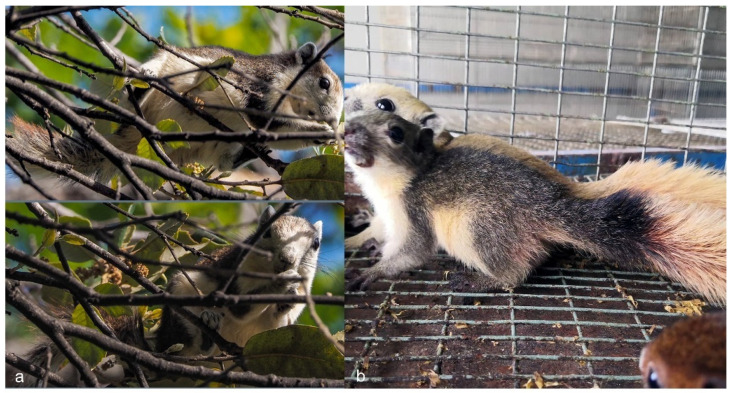
(**a**) Finlayson’s squirrel, *Callosciurus finlaysonii*, in a natural area in the province of Potenza, Italy. Photo by “golfopolikayakl” on i-naturalist.org (https://www.inaturalist.org/observations/79846755 accessed 1 October 2021). (**b**) Three Finlayson’s squirrels for sale by a pet store in the province of Frosinone (Italy): the white squirrel sold for €200, while the other two for €175. Photo downloaded from the store’s public Facebook profile.

Finlayson’s squirrel has been released in three countries outside its native range (Italy, Japan and Singapore), for a total of four introduction events, which all resulted in the establishment of a population [45] (Table 1 and Figure 2).

The invasion success of the species is in line with Bertolino [32], who estimated for a couple of *Callosciurus* a likelihood ratio of 0.73 to successfully establish a viable population after release (up to 0.90 if >four animals).

### 3.3. Pathways of Introduction

Squirrels are a large and charismatic group of mammals [46], whose appeal to people makes them commonly present in private houses and zoos [34]. Their trade, and subsequent release or escape, is their main pathway of introduction worldwide [32]. This is also the case of *Callosciurus* squirrels (Table 1). The first introduction of *Callosciurus* squirrels dates back to 1935 in Japan when some Pallas’s squirrels escaped from the zoological gardens on Izu-Oshima Island, Tokyo [69]. Since then, several other releases of pets and translocations have led to the establishment of 12 populations along the Pacific Coast in Central and Western Japan [57,63]. In Hong Kong—China, *Callosciurus* squirrels used to be sold in markets as pets or for their economic value (Chinese medicine or fur) [70]. Escaped individuals, or those deliberately released, of Pallas’s squirrels established populations during the late 1960s to the early 1970s [70]. In France, during the same years, a very small number of Pallas’s squirrels were imported directly from Asia by a single person (Chapuis & Pisanu, pers. comm.), and their release/escape led to the establishment of the initial French population in the city of Antibes [71]. Argentina is an emblematic case of how the perception of a charismatic alien species, such as the Pallas’s squirrels, can enhance the invasion of an IAS in the area of introduction. The first and only known introduction of Pallas’s squirrels in Argentina occurred in 1970 when a European family brought with them 10 individuals bought at a pet shop in the Netherlands, and 3 years later, two to five of them were released/escaped their ranch in Lujàn in the province of Buenos Aires [67,72]. Since then, Guichón at al. [67] recorded 26 translocations taken from the first invasion focus in Lujàn and another five translocations from three second invasion foci where squirrels were either released (*n* = 1) or kept in captivity (*n* = 4) [54]. In 1998, a number of Pallas’s squirrels escaped from a local animal trader in the area of Weert in the Netherlands, but their presence was confirmed in the region only in 2008 [73]. In Belgium, some squirrels probably escaped an abandoned zoo or a pet shop in Dadizele before 2005, when damage by squirrels was first recorded [61]. Finally, the origin of the Italian Pallas’s squirrel population is uncertain. Mazzamuto et al. [55] hypothesized that at least one pair of squirrels could have been released or escaped from a private house. In fact, the village in the province of Varese where the species was first recorded is known for a large number of vacation houses belonging to people from Belgium and the Netherlands. However, the possibility of an independent introduction through pet trade cannot be discarded [74].

Even if Finlayson’s squirrels have a small history of introductions worldwide compared to their congeneric Pallas’s squirrels, they follow the same path. At the moment, no information is available for the introduction of Finlayson’s squirrels in Singapore [45]. However, the two independent introductions recorded in Italy are both attributed to the animal pet trade and subsequent escape/release [45] (Figure 2). The population in Northern Italy originated in 1981 from two pairs of squirrels released in an urban park [75], while the population in Southern Italy originated through the release of three to four pairs in the mid-1980s [76]. In Japan, the presence of *C. finlaysonii* was detected by Oshida et al. [77] and confirmed by Kuramoto et al. [78] during genetic studies. However, it has not been confirmed if the squirrels in Hamamatsu are individuals of Finlayson’s squirrels sold in the pet market that were then released or escaped or hybrids of *C. erythraeus* and *C. finlaysonii*, possibly as a result of mixed-species cages in pet stores [77,78]. Further studies are needed to clarify the origin of this population.

## 4. Origin and the Challenge of Species Identification

A critical first step in invasion biology is the accurate identification of the invading taxon to evaluate the risks to which the area of introduction is exposed to in order to identify the potential pathways of introduction and prevent further introduction events and to assess the potential spread dynamics of the introduced species (e.g., [79,80]). The identification of an introduced species can be more challenging, because its population, generally generated by a few founding individuals with a high inbreeding rate, will likely differ, both genetically and phenotypically, from the parent population from which it is derived [81,82,83].

As previously mentioned, species and subspecies identification in *Callosciurus* squirrels, even in their native range, is sometimes challenging (e.g., [84]). Their classification has been mostly based on morphological features, such as pelage color, pattern and baculum’s shape and size [47,48]. However, for example, Boonkhaw et al. [85] showed how the pelage colors of Finlayson’s squirrels in Thailand do not consistently correspond to their genetic groups, suggesting that the genes associated with color may have variations and polymorphisms within subspecies. Balakirev and Rozhnov [56] found a similar result in their study: in Eastern Indochina, the morphological variability of the squirrels within the *C. erythraeus/finlaysonii* complex does not correspond to the phylogeographic pattern. Therefore, an integrative approach to taxonomy, where molecular markers and morphological features are complementary, could help in better identifying the *Callosciurus* species [55,56,85].

In Japan, three studies tried to identify or confirm the origin of Pallas’s squirrels. Oshida et al. [77] and Kuramoto et al. [78] investigated the origin of the population in Hamamatsu, Japan using mtDNA sequences. They confirmed what was previously hypothesized by Tamura [86] and Abe [87] that the Japanese population originated from Pallas’s squirrels from Taiwan. However, both research teams detected some specimens more closely related to *C. finlaysonii*, and Kuramoto et al. [78] attributed those genetic sequences to *C. finlaysonii* from Thailand. Oshida, Kuramoto and coworkers believed that their results could be related to hybridization or gene introgression (common results of closely related species such as *C. erythraeus* and *C. finlaysonii* kept in same small cages), while an alternative hypothesis is that some individuals of Finlayson’s squirrels sold in pet markets may have been released or escaped. Ikeda et al. [88] studied the *Callosciurus* population in the Japanese Kumamoto Peninsula, and their results also confirmed the Taiwanese origin of those Pallas’s squirrels.

In Argentina, Gabrielli et al. [54] compared mtDNA sequences from *Callosciurus* squirrels captured in Argentina to those of native and introduced populations in Asia. They found that the Argentinian squirrels were genetically closer to *C. finlaysonii* from an introduced population in Japan [77] than to *C. erythraeus*. This was another example of how the complex taxonomy of *Callosciurus*—in particular, that of the sister species *C. erythraeus* and *C. finlaysonii*—requires revision. They concluded that the species of *Callosciurus* in Argentina can be identified as *C. erythraeus*, because the distances between the sequences attributed to *C. erythraeus* and those from Argentina were comparable to the genetic intraspecific variations among sequences of *C. erythraeus* belonging to different subspecies or collected in different regions [54].

Three of the European populations of *Callosciurus* squirrels were investigated with a integrative approach to taxonomy where the mtDNA, pelage color and pattern and skull and mandible morphology were compared to a wide morphological and molecular dataset assembled for *Callosciurus* [55,74]. These data revealed a close similarity between French squirrels and Pallas’s squirrels from Taiwan, China. The populations from Italy and Belgium were similar to each other in their morphological and genetic characteristics and close to the specimens assigned to *C. erythraeus* (see, also, Reference [61] for the Belgian population). These results may indicate a common origin for the populations found in Belgium and Italy. However, some clear differences in their mandible morphology could indicate that these two populations may have shared the same native lineage but originated from independent introduction events [74]. Extending the investigation to nuclear markers could likely contribute to clarifying this issue (e.g., [61]).

Tamura et al. [89] tested a new tool for the identification and discrimination of six *Callosciurus* squirrels that could be used in integrative taxonomy. They showed how mating calls are species-specific and easily distinguishable and, hence, are a sensitive trait that can be used as an indicator of phylogenetic relationships. However, once again, this was not the case for Pallas’s and Finlayson’s squirrels, whose calls did not differ, confirming the monophyletic characters of these two species [56].

## 5. Early Detection and Effective Monitoring of IAS

When the prevention of an IAS introduction fails, the early detection of the species is key for a rapid and effective response [90]. However, if countries do not have a systematic monitoring system in place for their natural and semi-natural areas, the detection of the species often occurs when its presence and detrimental effects become visible because of an increase in the number of individuals. At this stage, the next step is to identify the distribution of the species using the most efficient and effective survey techniques and develop a management strategy [91].

Pallas’s and Finlayson’s squirrels are known for their activity of bark-stripping but, also, for cable or pipe gnawing (see the section on impacts for more details). In several countries where they were introduced, their presence was detected once these damages became evident (e.g., [61,62]). However, some studies showed the possibility of detecting the species even when at low densities after a new introduction/translocation event or after removal programs, when only a few individuals are left. In Japan, Tamura et al. [63] used sound playback surveys to detect Pallas’s squirrels during the first phase of settlement. Pallas’s squirrels use vocalizations during mobbing events or the mating period that attract conspecifics [92,93]. Using recordings of such acoustic signals, Tamura and colleagues were able to detect squirrels that would respond to those vocalizations or would become visible because of being attracted to the calling site. Another tool that was successfully used in detecting squirrels was camera traps. Once deployed, camera traps need only periodical checks to replace the batteries; SD cards or the bait used as an attractive trap for the squirrel (nuts, seeds and nut spread); hence, they are an efficient monitoring tool, especially in areas with a low density of squirrels ([61]; Mazzamuto, pers. comm.).

A common tool used to detect squirrels and also used for Pallas’s squirrels is a hair tube [94,95]. These segments of PVC pipes with sticky tape applied to the internal ends of the tubes are placed along tree branches or tree trunks. Once the squirrel walks through the tube attracted by the bait inside, some of its hairs will stick to the tape; those hairs can then be analyzed and identified to the species [96]. Hair tubes were successfully used both in Italy and Argentina to monitor the presence and expansion of Pallas’s squirrels ([97], Mazzamuto, pers. comm). Moreover, in Southern Italy, Ancillotto et al. [98] assessed the effectiveness and efficiency of hair tubes versus visual surveys in the detection of Finlayson’s squirrels. While both sampling methods were successful, hair tubes resulted in fewer false absences and were 33% less expensive than visual sampling.

Visual surveying was the monitoring technique used in almost all the areas of introduction of *Callosciurus* squirrels. During these surveys, the operators, walking along transects or visiting different wood patches, aimed to visually detect the squirrels, to hear their calls or to detect signs of their presence such as nests (especially in those areas where there were no native squirrels) or bark-stripping [61,62,63,71,98,99]. In France, the distance sampling of individuals and nests along line transects produced reliable estimates of the relative abundance of Pallas’s squirrels in different habitats [71].

Visual surveys were often integrated with interviews of citizens and stakeholders. The diurnal habit of these tree squirrels, their vocalizations and their activity of bark-stripping makes them also detectable to nonexpert eyes. Interviews and sighting reports have proven to be very effective in Argentina, where residents have highly contributed to the detection of new populations and the reconstruction of the history of their introduction [67,99,100]. However, Guichón et al. [67] highlighted that there was a lag in the time between the detection by residents of Pallas’s squirrels in a new area and when they reported this observation. This time lag was reduced by increasing the communication activities that helped in changing people’s perceptions on this species, from charismatic to a dangerous species for the native environment and human activities. In other areas of introduction of both *Callosciurus* species, interviews were effective not just to detect the presence and expansion of the population (e.g., [62,76]) but, also, to detect the last individuals left during eradication programs [61,62].

## 6. The Establishment and Spread of IAS

Both species of *Callosciurus* are, as already mentioned above, habitat generalists, and their social system of strongly overlapping home ranges allows them to reach a high density in a variety of habitat types. For *C. erythraeus*, their density in Taiwan (native range) was estimated at 4.3 animals/ha in tropical monsoon forests, while most densities documented for populations in the introduction areas were even higher (5.6/ha in a fragmented area in Belgium [61]. 6.8/ha in an evergreen tropical forest in Japan [101] and 7.8/ha in mixed forests in Italy [102]; the standard errors of these estimates were not provided). These high densities and high reproductive rates (with even three litters per year for some females and a maximum number of nine uterine scars per year for females from the Italian population [103]) are prerogatives for a good expansion capacity. As for most invasive mammal species, the initial expansion (first decades after the first introduction(s)) is slow, speeding up with the increasing abundance of the populations. For example, in the main area of occurrence in Argentina, the increment of radial distribution was estimated at 0.3 km/year in 1973–1999, while it increased to 1.6 km/year in the following 5 years (1999–2004 [104]). This increase was accompanied by only a slight change in perception on the part of the inhabitants, from purely positive as an attractive animal (even for tourists) to a pest causing different types of economic damage. By 2013, this main invasion area extended over >1300 km^2^, with an estimated abundance of 100,000 individuals [99]. Moreover, the frequent translocations, linked to the high appeal of the species, resulted in other invasion areas in various phases of the expansion speed [66,99]. These illegal translocations are probably much more frequent than those documented and help to explain the expansion speed for this species, which has a limited gap-crossing capacity and short perceptual range, even in fragmented landscapes [105]. Another example from the population introduced in the city of Antibes in France at the end of the 1960s calculated an exponential range increase between 1995 and 2015, even if the diffusion coefficients were low (0.08–0.20 km/year [71]). Additionally, in its distribution area in France, the majority of the public appreciates Pallas’s squirrels, especially in the urban and suburban areas, where many inhabitants have never seen native red squirrels.

A similar expansion trend was observed for the *C. finlaysonii* population in South Italy that originated from three to four pairs released in the mid-1980s and, by 2005, already occupied 26 km^2^ [76]. The current observed distribution and the predicted future one suggest a further range expansion that will bring this IAS into contact with the endemic Calabrian black squirrel (*Sciurus meridionalis*), demanding urgent and drastic control of the alien species [106,107]. Hence, knowing the attitudes of the local public and stakeholders about the presence of this IAS, planning its management would be extremely useful.

Finally, to decide, prioritize and plan management strategies and actions, integrating climate change and land use change scenarios in order to model the future spread of invasive squirrels is paramount [107,108]. Considering both parameters, species distribution models for alien squirrels in Italy have predicted a potential loss in suitable habitats and in dispersal corridors, limiting their future range expansion [107].

## 7. Impacts of IAS on Environment, Economy and Human Health

As part of the definition, an invasive species introduction causes environmental or economic damage or harm to human health [109]. *Callosciurus* squirrels introduced worldwide fit this description, causing harm to native species, damaging people’s properties and affecting human activities and, potentially, affecting human health.

### 7.1. Harm to Native Species

Pallas’s squirrels are suspected to affect native squirrel species through interspecific competition in Japan, where it is in syntopy with the Japanese squirrel *Sciurus lis* [69,110], and in France and the Netherlands, where it cooccurs with the Eurasian red squirrel *S. vulgaris* [62,71]. In these two European countries, researchers and citizens have reported a lower number of red squirrel observations, mainly at the periphery of the area on which Pallas’s squirrels have become established. In Italy, Mazzamuto and coworkers specifically described the competition mechanism between Pallas’s squirrels and Eurasian red squirrels and the outcome of interspecific interactions [102,111]. The native and invasive species have a high degree of niche overlap and compete for space and food resources [111]. Red squirrels, when in cooccurrence with the IAS, are distributed in patches and increase the intraspecific space overlap up to three times [111]. There is no niche partitioning in space use, since the home ranges of the two species overlap considerably. Moreover, native squirrels have a relatively poorer body condition in areas with, compared to areas without, Pallas’s squirrels with reduced juvenile growth and adult body mass, possibly caused by faster food depletion by the IAS, which lives at much higher densities than the native species in the areas of cooccurrence (7.8 animals/ha against the 0.05 animals/ha for red squirrels or, on average, 150 Pallas’s squirrels for every single red squirrel) [102,111]. Another hypothesis for the poor body condition is that red squirrels may avoid good-quality forest patches occupied by Pallas’s squirrels and shift to poor-quality habitats in terms of tree species diversity, food availability and/or degree of fragmentation, having an adverse effect on the population demography [111]. In fact, the ongoing eradication program of Pallas’s squirrels in Italy has shown that the removal of invasive squirrels in some areas allows the return of the red native squirrel, who is probably relegated to the borders of the distribution area of Pallas’s squirrels [102]. The competition between the two species leads to a dramatically low local survival and a consequent low density of native red squirrels (8–14 times lower than in areas without Pallas’s squirrels) [102]. The final outcome of this process is the replacement of the native squirrel by the invasive one, as for the better-known case of the Eurasian red squirrel and the introduced invasive Eastern grey squirrel in Europe [112].

Other native wildlife that could be affected by the presence of the *Callosciurus* squirrels is the bird community. In Argentina, Pallas’s squirrels affect the bird species richness, suggesting a species-specific response of the avian community in an area where native arboreal diurnal mammals are not present [113]. Pallas’s squirrels did not systematically consume bird eggs (see, also, Reference [114] in Argentina and [115] in Japan), but Messetta et al. [113] suggested that squirrels might interfere with birds that modify their behavior, affect reproductive success or compete for food and nesting sites.

*Callosciurus* squirrels represent a threat to forests as well. One of the first visible signs of the presence of *Callosciurus* squirrels is bark-stripping. The bark removal of trees and shrubs can reduce the resistance of the plant to parasites and mechanical stress, ultimately affecting its survival and, thus, altering the forest composition. Both in their native and introduced ranges, bark-stripping by Pallas’s squirrels is related to nutrition and nest construction [116,117,118,119,120,121]. Bark-stripping has been reported in most of the countries where Pallas’s and Finlayson’s squirrels have been introduced [61,62,71,122,123]. In Argentina, Pallas’s squirrels have debarked 40% of the trees of the area investigated in the Pampas region and 88% of the tree and shrub species present [123]. In Italy, Finlayson’s squirrels have affected 79% of the trees in Northern Italy and 51% in Southern Italy through bark-stripping, jeopardizing the survival of the affected trees or parts of them [122]. The plant species are not specifically selected, and their use is based on their availability [122]. Despite the localized distribution in Italy, these authors suggest that, in Italy, the bark-stripping damage caused by Finlayson’s squirrels might be higher than that by grey squirrels [122,124].

*Callosciurus* squirrels might also alter the forest composition through seed dispersal. The ability to consume novel food, opportunistic food choices and an overall feeding plasticity are characteristics that allow *Callosciurus* squirrels to establish themselves in natural and modified habitats [36,114,117]. Pallas’s squirrels have been described as seed dispersers in their native and introduced ranges through seed hoarding and epi- and endozoochory [86,125,126,127,128]. In Argentina, the germination in a laboratory of seeds collected from Pallas’s squirrel feces was recorded for two exotic trees, *Morus alba* from China and *Casuarina* sp. from Australia, which confirmed the potential role of Pallas’s squirrels as seed dispersers [128]. In the same study, Bobadilla and coworkers [128] assessed the maximum size for the passage of entire seeds through the digestive tract, information that, considering the wide feeding plasticity of the species, can help in predicting its role as a potential disperser or predator of other species in other invaded communities.

### 7.2. Parasites, Zoonosis and Human Health

#### 7.2.1. Macroparasites

Passing through the pet trade, anti-parasite treatments of captive animals and the stochastic effects related to small founder population sizes are likely to contribute to the loss of many species of the original parasite community of alien species (e.g., [129]). This so-called “Enemy Release Hypothesis” (ERH) is often invoked as one of the mechanisms contributing to successful invasions [130,131]. Other ecological processes related to the parasites of IAS are disease-mediated competition (DMC) and parasite-mediated competition (PMC), which can exacerbate the ecological impact of alien tree squirrels on native species. A paradigm is the Squirrelpox virus (SQPV) transmitted by invasive grey squirrels, that act as healthy vectors, to native red squirrels, for which the disease is lethal in the vast majority of cases (e.g., [132]). As far as macroparasite-driven PMC is concerned, a spillover of an alien nematode (*Strongyloides robustus*) from grey squirrels, introduced in Italy, to the native red squirrels reduces the survival in infected animals with high parasite loads [43].

To date, there has been no evidence for DMC or PMC connected to the introduction and subsequent spread of squirrels of the genus *Callosciurus*, but only a few studies have monitored their parasite fauna.

The parasite species richness of *C. erythraeus* in its native range was recently reviewed by Gozzi et al. [130]. They calculated a richness (S) of 33 species of macroparasites and compared it with the much lower numbers found in several of the non-native populations (Italy S = 11, Japan S = 9, Argentina S = 6 and Belgium and France S = 3), strongly suggesting that the loss of many parasite species has contributed to the invasion success of this species in most, if not all, of the introduction areas [130].

In the past decade, there has been growing interest in microparasite infections and the macroparasite fauna of *Callosciurus* in its non-native range. Introduced Pallas’s squirrels carried few species from their original macroparasite community with them to the non-native ranges (Table 2). This was observed mainly in populations in Japan, where the helminth *Strongyloides callosciureus* was successfully co-introduced with its original host [133,134] while other *Callosciurus*-specific nematodes, *Brevistriata callosciuri* and *Gongylonema neoplasticum*, were detected in only one to three hosts [134,135,136]. In Argentina, France, Belgium and Italy, the introduction of *Callosciurus*-specific parasites was detected to a very low extent, possibly resulting in extinction of the parasite in the future [130,131,137,138,139]. Consequently, in these countries, there is a very low risk of macroparasite spillover from the alien to native rodent species. Since, so far, Pallas’s squirrels are only accidentally parasitized by nematodes acquired in their new environment (Table 2), the risk of parasite spillback also seems to be very low (France and Belgium [137], Italy [131] and Argentina [138,139]).

The situation with ectoparasites might be more complex and potentially more dangerous for the emergence of zoonotic diseases. Shinozaki et al. [140] found fleas (*Ceratophyllus (Monopsyllus) anisus*) and four nymphs of the tick *Haemaphysalis flava* (on a single animal) in a population on Honshu Island, Japan, suggesting that further studies are needed to assess the risk of transmission to humans of plague by the fleas and of tularemia and Japanese spotted fever (*Rickettsia japonica*) by the ticks. Another example of a successful co-introduction is that of the sucking louse *Neohaematopinus callosciuri*, found at a relatively high prevalence in a non-native population in Japan [141]. The possible spillover of this louse to other (native) hosts was not investigated, nor the risk of the zoonotic transmission of louse-borne typhus [140]. Data from Italy suggest that ectoparasites, both generalist species (the hard tick *Ixodes ricinus*) and species typical of the native red squirrel (the flea *Ceratophyllus sciurorum sciurorum*), can be easily acquired by Pallas’s squirrels, increasing the risk of spillback processes and an overall increase in the parasite populations, with potential consequences for disease transmission and a potential risk for human health (e.g., *Borrelia burgdorferi* transmitted by hard ticks infesting alien sciurids [131,142]).

Whether *Callosciurus* squirrels host zoonotic macroparasite needs further studies. In Italy, a case of two pet *C. prevosti* both infected with the cestode *Hymenolepis diminuta*, known also as rat tapeworm, was reported by d’Ovidio et al. [143]. Only a few hundred cases of human infections by this species have been recorded, almost exclusively in children: although human hymenolepiasis is usually asymptomatic, sometimes severe symptoms occur (for details, see Reference [143]).

To our knowledge, there are no studies of Finlayson’s squirrel parasites in their areas of introduction. However, a study of anatomopathological lesions found on *C. finlaysonii* squirrels in Southern Italy revealed intestinal, renal and hepatic lesions [144]. The authors suggested the hypothesis of a metabolic disorder caused by a diet not suitable for this alien species, but no reference to lesions attributable to parasites was made [144].

#### 7.2.2. Microparasites

IAS can act in their non-native range as reservoirs for emerging infectious diseases, often caused by microparasites (viruses, bacteria, fungi and protozoa). They can also become infected with already existing microparasites, becoming new reservoirs and enhancing pathogen transmissions. Hence, IAS can potentially spread diseases to native wildlife and to man.

In recent years, several studies have searched for the occurrence of microparasites in some of the native (e.g., [146,147]) and non-native populations of *C. erythraeus* or *C. finlaysonii*. For example, recently discovered variegated squirrel bornavirus 1 (VSBV-1) was detected in five different squirrel species held in captivity (among which was *Callosciurus prevostii)* and was associated with cases of fatal encephalitis in their caretakers [148]. In the same study, two novel herpesviruses and one of polyomavirus were recently described in the Italian population of Pallas’s squirrels [148]. Since they were detected only in this host species, it is likely they are all species-specific and that Pallas’s squirrels are their original hosts [148]. Another recent study investigated the presence of leprosy bacteria *Mycobacterium leprae* and *M. lepromatosis* in the Italian population of Pallas’s squirrels, but no DNA from leprosy bacilli was detected in any of the samples tested. The transmission of protozoan microparasites to other hosts, mainly native red squirrels, seems also unlikely: two very detailed studies on *Eimeria* spp. and *Cryptosporidium* spp. concluded that the latter was a different genotype than found in red squirrels, while, for *Eimeria*, two hypotheses remain open: a spillover of *E. sciurorum* from red to Pallas’s squirrels or the occurrence of a species recently acquired by the IAS [149,150].

In the population of Finlayson’s squirrels in Southern Italy, Iatta et al. [151] detected cryptococcosis, a fungal disease acquired from the environment of introduction, for which Finlayson’s squirrels may serve as sentinels for human exposure.

In conclusion, for the moment, these studies are descriptive, aiming at determining whether *Callosciurus* are hosts for certain microparasites. To reveal a potential role in parasite transmission to other wildlife and/or to humans, more detailed research is necessary.

### 7.3. Harm to Human Activities, Properties and Economic Impact

*Callosciurus* squirrels also negatively affect human activities, properties and, potentially, economy. Pallas’s squirrels in Argentina affect productive systems through their feeding activity and gnawing behavior, reducing fruit production in orchards, consuming cereals in silos and gnawing on pipes and cables that damage irrigation and cooling systems in cultivation and poultry farms [65,104]. However, the effective economic loss caused by the IAS has not been assessed. Similar effects have also been recorded on private properties where Pallas’s squirrels chewed on electrical cabling, such as lighting, telephone and television cables ([62,104]; Mazzamuto, pers. comm). However, the behavior of *Callosciurus* squirrels that can mostly harm a region’s economy is bark-stripping in plantations, which causes a loss for the wood industry and, also, carries additional costs due to the mitigation and control measures that are required. Pallas’s squirrels in their native range are known to severely damage tree plantations of *Cryptomeria japonica*, *Cunninghamia lanceolata*, *Pinus luchuensis* and *Pinus elliottii* [152]. This same issue has been reported in Argentina in plantations of *Pinus elliottii*, *Eucalyptus dunnii* and *Populus deltoides* [153]. The damage to the trees produces deformations in the section of the tree most relevant to the wood market (between 3 m and 6 m in tree height), causing a loss of 20–58% of the wood volume and/or a reduction in the tree height [153].

## 8. Management and Regulations: Failures and Successes

Generally, specific laws or even codified management strategies for introduced *Callosciurus* are nonexistent or minimal. The management of invasive species is an interdisciplinary endeavor that requires a collaborative approach and communication to ensure the integration of biological, social, political and economic insight [154]. Prevention is, of course, the preferred strategy [155]. Other than inaction, management strategies for established populations are generally focused on three options: prompt eradication, containment and population control [155]. Eradication may be appropriate in isolated and newly colonized areas. Theoretical assessments and empirical results from introductions demonstrate the viability of small populations of tree squirrels, and therefore, eradication must happen quickly [156].

In Argentina, there are no systematic management plans at the national level for the control of *C. erythraeus* (Guichón, pers. comm. [153]). Local population reduction at specific sites is the result of isolated and independent actions by local producers that suffer crop depredation attributed to this species [153]. Although illegal to sell and transport or release squirrels within the country over the past 25 years, efforts to control such detrimental actions have been unsuccessful [67]. The National Strategy on Exotic Invasive Species [157] now provides an appropriate framework to work with the national and provincial wildlife agencies; management protocols are being developed to control the invasive Pallas’s squirrel, at least in some provinces (Guichón, pers. comm.).

In Europe, *C. erythraeus* was inserted into the first list of ‘Invasive Alien Species of Union Concern’ (EU regulation 1143/2014) that are subject to a ban on import, trade, breeding and release and for which each EU member country where these species are established have the obligation to produce management plans (Genovesi et al. 2014). *Callosciurus finlaysonii* has not been added to the Union list yet, but its risk assessment is under review.

In Italy, despite the European Regulation and the existence of a national species management plan for Pallas’s squirrels, the major problem for the control or eradication of invasive squirrels is the lack of a hierarchical chain from planning and command to the actual execution of control/eradication actions, with much of the effort expected from local authorities with a high degree of autonomy together with low financial support and a lack of political interest (Wauters & Mazzamuto, pers. comm.). A lack of continuous effort directed towards control by trapping and euthanasia, as well as by shooting, has resulted in insufficient results in the control of Pallas’s squirrels [158]. Italy has no management plan for *C. finlaysonii*: the small population at Acqui Terme has never been subject to control, while the Southern Italian population around the town of Maratea has only used low-intensity trapping, with carcasses of the euthanized animals used for a series of veterinary studies [144]. The impact of these limited removals on the population dynamics is unknown but appears negligible. Since EU Member countries may establish a list of Invasive Alien Species of National Concern, to which provisions and restrictions foreseen for the species of Union Concern may be applied at the national level, the Finlayson’s squirrel has been classified as an IAS whose management is a priority in Italy [159].

In France, a national plan for control was launched in 2011 [160] and implemented in 2012 in the Alpes-Maritimes (Antibes-Vallauris-Cannes) and in 2016 in the Bouches-du-Rhône (Istres). Pallas’s squirrels are controlled using two methods, shooting and trapping followed by euthanasia by neck dislocation. While, in the Bouches-du-Rhône, given the estimated population size (200–300 individuals in 2015) and its limited distribution area, eradication appears to be a realistic objective, in the Alpes-Maritimes, the species occupies an area too large for eradication to be possible; only population control is achievable [68]. Control actions for the period 2011–2014 were planned at about €100,000 per annum [160].

Proactive approaches to stem importation can be effective in minimizing impacts by assessing the risk of the invasiveness of a species yet to be seen in a country, an approach taken in some parts of Oceania and Asia [45]. New Zealand and Australia use a proactive ‘guilty until proven innocent’ approach [161] that bans all species, including *Callosciurus* squirrels, from importation, except for those for which a risk assessment has been completed and formal approval granted. Japan has a similar system: under law number 78 of the ‘Invasive Alien Species Act’ (Ministry of the Environment, Government of Japan, 2004: http://www.env.go.jp/nature/intro/index.html accessed 1 October 2021), *C. erythraeus* is forbidden from being bred, transported and imported, and its populations must be controlled. *Callosciurus finlaysonii*, however, is designated as an Uncategorized Alien Species, and its import and keeping in Japan requires 6 months of investigation for a risk assessment.

Some have suggested the idea of ‘ecological citizenship’ for invasive species that are localized with a minimal risk for expansion or where the native species has been eliminated or replaced [162]; however, no political units have passed laws that have adopted this approach. Social perceptions and attitudes towards charismatic introduced species play a key role in determining the vector activity. Their appeal to humans is the reason for repetitive transport and release into new sites and also the reason for the lack of support of control actions by various social groups [66,163]. Such is the case in Argentina, where incipient populations were highly valued by the local residents and often translocated and provided supplemental food. As a result, a challenge is that prevention and rapid response need to be implemented when and where no urgent or obvious impacts are evident to the local people [67].

The only successful eradication of *Callosciurus* in Japan occurred following the detection of <10 animals in Kanagawa, while efforts against other populations have been ineffective [63,164]. In Europe, two out of four countries were successful in eradicating Pallas’s squirrels. In Belgium, an eradication campaign using baited mesh wire life traps took over 5 years. The removal of 248 squirrels required an investment of over €200,000, including 1.5 years of post-eradication surveying [62]. In the Netherlands, the key to success was an intensive effort of trapping, transportation to a specialized captive animal center and sterilization of 250 squirrels that cost €330,000 (pers. comm., M. La Haye [63]). This strategy was adopted thanks to the restricted size and range of the population (e.g., the Genoa Nervi population of Eastern grey squirrels in Italy [165]). In both countries, the support of the public and private landowners that allowed access to their land and reported sightings was considered one of the most important ingredients for success [62,63].

## 9. Conclusions

Given the unique nature of the conditions that permit total eradication, the containment of established populations that cause ecological or economic damage in a space may be achievable through early detection and the effective use of habitat barriers. However, population control programs may be the only true alternative to inaction in the vast majority of cases of *Callosciurus* invasions [45]. Intensive live trapping with euthanasia, sterilization with release or retention in captivity and shooting are the methods used with maximum success in the eradication of small, localized populations or population control in Belgium, France, Italy, Japan and the Netherlands [62,63,158,160,164]. For any management actions, success is due to a broad-based socioecological approach where human interactions are given consideration [166]. Communication with the public and transparency about these activities are critical to the success of such efforts in nearly all the examples of eradication, especially in urban areas [62,63,67,160]. Efforts to educate and involve the broader public by actively engaging a diversity of stakeholders are more likely to build a consensus and should be a priority [45]. The uses of citizen scientists can increase engagement, facilitate the understanding of scientific data and create a vested interest in the success of management efforts [167]. Ethical, legal, economic and scientific considerations must inform these efforts in order to expeditiously approach a consensus on the critical management efforts used in the biological invasions of “beautiful squirrels”, for beauty is in the eye of the beholder.

## Figures and Tables

**Table 1:** Summary of the number of releases (introductions and translocations) of *Callosciurus* squirrels in the world.

Country	Year First Introduction	Pathway	Releases	Established	Not Established/Rare	Removed/Eradicated
*Callosciurus erythraeus*						
Japan	1935	Zoo	20	13	6	1
China—Hong Kong	1960s–1970s	Pet trade	2	2		
France	1960s–1974	Pet trade	2	2		
Argentina	1970	Pet trade	28	23	5	
The Netherlands	1998	Pet trade	1			1
Belgium	<2005	Zoo/Pet trade	1			1
Italy	<2011	Pet trade	2	1	1	
*Callosciurus finlaysonii*						
Japan	1970	Pet trade	1	1		
Italy	1980s	Pet trade	2	2		
Singapore	-	-	1	1		

Update of Bertolino and Lurz [45]. New literature included: Tamura et al. [63] (Japan), Chapuis et al. [68] (France), Guichón et al. [67] (Argentina), La Haye [62] (the Netherlands), Adriaens et al. [61] (Belgium) and Mazzamuto et al. [55] (Italy).

**Table 2:** Macroparasites of Pallas’s squirrels in their areas of introduction.

Species	Country	Parasite/Host Relationship	References
**Endoparasites**			
*Brevistriata callosciuri*	Japan	Specific	Matsudate et al. [135]; Miyabe et al. [134]
Capillariinae	Italy	Acquired	Mazzamuto et al. [131]
	Japan	Acquired	Miyabe et al. [134]
*Gongylonema neoplasticum*	Japan	Specific	Asakawa [136]
*Hymenolepis* sp.	France	Acquired	Doziéres et al. [137]
*Mastophorus* sp.	Belgium	Acquired	Doziéres et al. [137]
*Pterygodermatites* sp.	Argentina	Acquired	Gozzi et al. [138]
*Rictularia cristata*	Japan	Acquired	Miyabe et al. [134]
*Rodentoxyuris sciuri*	Italy	Acquired	Mazzamuto et al. [131]
*Spiruridae*	Italy	Acquired	Mazzamuto et al. [131]
*Stilestrongylus* sp.	Argentina	Acquired	Gozzi et al. [138]
*Strongyloides callosciureus*	Italy	Specific	Mazzamuto et al. [131]
	Japan	Specific	Sato et al. [133]; Miyabe et al. [134]
*Strongyloides* sp.	Italy	-	Mazzamuto et al. [131]
	Japan	-	Matsudate et al. [135]
*Trichuris muris*	Italy	Acquired	Mazzamuto et al. [131]
**Ectoparasites**			
*Androlaelaps fahrenholzi*	Argentina	Worldwide	Benitez et al. [65]
*Ceratophyllus s. sciurorum*	Italy	Acquired	Mazzamuto et al. [131]
*Cheyletus* sp.	Argentina	Worldwide	Gozzi et al. [130]
*Ctenophtalmus agyrtes*	Italy	Acquired	Mazzamuto et al. [131]
*Ctenophtalmus* sp.	Italy	Acquired	Mazzamuto et al. [131]
*Cuterebra* sp.	Argentina	Acquired	Gozzi et al. [130]
*Enderleinellus kumadai*	Belgium	Specific	Doziéres et al. [137]
	France	Specific	Doziéres et al. [137]
	Japan	Specific	Durden & Musser [145]
*Haemaphysalis flava*	Japan	Specific/Acquired	Shinozaki et al. [140]
*Hoplopleura erismata*	Belgium	Specific	Doziéres et al. [137]
*Ixodes ricinus*	Italy	Acquired	Mazzamuto et al. [131]
*Monopsyllus anisus*	Japan	Specific/Acquired	Shinozaki et al. [140]
*Neohaematopinus callosciuri*	Japan	Specific	Shinozaki et al. [141]
*Nosopsyllus fasciatus*	France	Worldwide	Doziéres et al. [137]
*Ornithonyssus* cf. *bacoti*	Argentina	Worldwide	Gozzi et al. [130]
*Polygenis rimatus*	Argentina	Acquired	Gozzi et al. [130]; Gozzi et al. [139]
Trombiculidae	Italy	Acquired	Mazzamuto et al. [131]

Update of Gozzi et al. [130,138]. New literature included: Mazzamuto et al. [131], Miyabe et al. [134] and Gozzi et al. [139].

## Data Availability

Not applicable.
